# Treatment outcome of nimotuzumab plus chemotherapy in advanced cancer patients: a single institute experience

**DOI:** 10.18632/oncotarget.8516

**Published:** 2016-03-31

**Authors:** Shuping Xu, Mayra Ramos-Suzarte, Xianhong Bai, Binghe Xu

**Affiliations:** ^1^ Department of Medical Oncology, Cancer Hospital, Chinese Academy of Medical Sciences and Peking Union Medical College, Beijing, P. R. China; ^2^ Department of Medical Affairs, Biotech Pharmaceuticals Co., Ltd., P. R. China; ^3^ Department of Clinical Research, Center of Molecular Immunology, Havana, Cuba

**Keywords:** nimotuzumab, monoclonal antibody, chemotherapy, advanced cancer

## Abstract

Nimotuzumab is a humanized anti-EGFR IgG1 monoclonal antibody and demonstrates a better safety profile than other anti-EGFR antibodies due to its intermediate affinity. Since it was approved in China for the treatment of nasopharyngeal cancer (NPC), it has been widely used in NPC and in many clinical trials for other cancer types. However, the optimal dose and administration frequency of nimotuzumab that should be used and which kind of cancer patients will be more benefited from nimotuzumab is still unknown. In this retrospective study, 205 advanced cancer patients with colorectal cancer, esophageal cancer, head and neck cancer, gastric cancer, non-small cell lung cancer, or other cancers from mainland China, treated with nimotuzumab in combination with chemotherapy, were enrolled. Over 60% of these patients received nimotuzumab > 6 doses and ≥ 400 mg/week as maintenance therapy. It was well tolerated in real-life patients. This report demonstrates that age, sex and previous treatment might be potential predictive factors for survival, and patients received nimotuzumab > 6 doses and > 200 mg/week might benefit more from nimotuzumab therapy. Using these factors for stratification analysis may form a predictive differential clinical strategy for nimotuzumab to maximize the benefit in patients with different epithelial tumors.

## INTRODUCTION

Epidermal Growth Factor Receptor (EGFR [HER-1, erbB1]), a transmembrane glycoprotein, is a receptor widely expressed on a variety of tissues such as skin, gastrointestinal tract and has activity in the signaling pathway promoting cell growth, differentiation, proliferation, and inhibition of apoptosis [1, 2]. However, there is well-documented evidence that up-regulation of the EGFR signal transduction pathway is involved in the establishment and spread of tumors of epithelial cell origin [3–5]. EGFR is dysregulated in several malignant tumors located in head and neck, esophageal, gastric, lung, colorectal, and other organs [6], which correlates with increased metastasis, decreased survival, a poor prognosis [7–10] and radiotherapy (RT) and chemotherapy (CT) resistance[11, 12]. Thus, agents that bind to EGFR and inhibit the EGFR pathway would be expected to exert antagonistic biological activity [6, 13]. Currently, EGFR tyrosine kinase inhibitors (gefitinib, erlotinib, lapatinib) and anti-EGFR monoclonal antibodies (cetuximab, nimotuzumab, panitumumab, and matuzumab) have been developed for the treatment of different malignancies.

Nimotuzumab (alternatively referred to as TheraCIM®, Theraloc®, CIMAher®, BIOMAb-EGFR®, Tai Xin Sheng®, OSAG-101 or YMB-1000) is a humanized IgG1 monoclonal antibody targeting the extracellular domain of EGFR. It has demonstrated blocking ability against the binding of EGF and TGF-alpha to EGFR, and has observed inhibitory activity on tumor cell growth, angiogenesis, and apoptosis [14–16]. Further, experimental observations demonstrated that in contrast to other approved anti-EGFR antibodies, the intrinsic properties of nimotuzumab require bivalent binding for stable attachment to the cellular surface, leading nimotuzumab have the maximum clinical benefit and absence of severe dermatological toxicity (high uptake in tumors overexpressing the receptor and low uptake in normal tissues) [17–23]. It has been approved for the treatment of advanced head and neck cancer (H&NC) [24–26], nasopharyngeal cancer (NPC) [27], glioma [28, 29] and esophageal cancer (ESOC) [30] in 30 countries.

Nimotuzumab (trade name in China Tai Xin Sheng®, Registration ID: 2005S02236) was approved in China in 2008 as a drug in combination with RT for a treatment of NPC and was included within Chinese NCCN guideline as a recommended targeted therapy for this indication in 2009.

Post marketing experience in NPC reinforces the safety within Chinese population [31–33]. More than 30,000 patients received this therapy with an excellent safety profile in China [34] and throughout the world [21, 35, 36]. Five phase III clinical trials are ongoing in different tumors from epithelial origin with different schedules of treatment, with the approval of the China Food and Drug Administration (CFDA). For this reason, physicians have used nimotuzumab as an “off-label product” in other cancers of epithelial origin.

After seven years of the first approval in China, the information of several advanced cancer patients who received nimotuzumab in combination with CT in off-label approach has been collected. This retrospective analysis summarizes the safety profile, efficacy and possible predictive factors of this anti-EGFR therapy in Chinese patients with advanced cancers.

## RESULTS

### Patients' characteristics

Comprising our retrospective study were 205 cancer patients with various diagnoses. Table 1 shows the distribution of patients by tumor-type and schedule treatment. Colorectal cancer (CRC), ESOC, H&NC, gastric cancer (GC), non-small cell lung cancer (NSCLC) and other cancer patients (which consisted of low numbers of breast, pancreatic, bile duct, gallbladder, renal pelvis and ovarian cancer) were included. Patients' characteristics are described in Table 2. In total, 139 patients were male (67.8 %). The majority of patients had stage IV disease (97.6%) when received nimotuzumab and 140 (68.3%) patients were adenocarcinoma (ADC). The 66.8% (137/205) of the patients were younger than 60 years of age. Moreover, 60% of patients underwent surgery, 43.9% (90/205) received RT/chemoradiotherapy (CRT), and 80.5% (165/205) had CT before nimotuzumab.

**Table 1:** Distribution of patients by tumor localization and schedule treatment

Nimotuzumab (mg/week)	100	200	250	300	400	500	600	Total (%)
n (%)	6 (2.9)	47 (22.9)	1 (0.5)	12 (5.9)	130 (63.4)	1 (0.5)	8 (3.9)	205 (100)
NSCLC	0	5	0	2	16	0	0	23 (11.2)
ESOC	0	11	0	1	8	0	1	21 (10.2)
CRC	0	12	1	6	48	1	3	71 (34.6)
H&NC	2	11	0	1	18	0	2	34 (16.6)
GC	3	3	0	1	27	0	1	35 (17.1)
Others	1	5	0	1	13	0	1	21 (10.3)

NSCLC: non small cell lung cancer; ESOC: esophageal cancer; H&NC: head and neck cancer; CRC: colorectal cancer; GC: gastric cancer.

**Table 2:** Patients demographic and tumor characteristics

Characteristic		Total	H&NC	CRC	ESOC	GC	NSCLC	Others
		n (%)	n (%)	n (%)	n (%)	n (%)	n (%)	n (%)
Sex	Male	139(67.8)	26(76.5)	48(67.6)	21(100)	24(68.6)	16(69.6)	4(19)
	Female	66(32.2)	8 (23.5)	23(32.4)	0	11(31.4)	7(30.4)	17(81)
Age (yr)	<60	137(66.8)	23(67.6)	46(64.8)	9(42.9)	23(65.7)	16(69.6)	20(95.2)
	≥60	68(33.2)	11(32.4)	25(34.7)	12(57.1)	12(34.3)	7(30.4)	1(4.8)
Histopathology	ADC	140(68.3)	2(5.9)	71(100)	0	33(94.3)	14(60.9)	20(95.2)
	Non-ADC	65(31.7)	32(94.1)	0	21(100)	2(5.7)	9(39.1)	1(4.8)
Clinical stage	III	5(2.4)	2(5.9)	0	1(4.8)	0	2(8.7)	0
	IV	200(97.6)	32(94.1)	71(100)	20(95.2)	35(100)	21(91.3)	21(100)
**Previous treatment**								
Surgery	NO	82(40.0)	21(61.8)	15(21.1)	11(52.4)	20(57.1)	13(56.5)	2(9.5)
	YES	123(60.0)	13(38.2)	56(78.9)	10(47.6)	15(42.9)	10(43.5)	19(90.5)
RT/CRT	NO	115(56.1)	8(23.5)	47(66.2)	5(23.8)	32(91.4)	13(56.5)	10(47.6)
	YES	90(43.9)	26(76.5)	24(33.8)	16(76.2)	3(8.6)	10(43.5)	11(52.4)
CT	NO	40(19.5)	18(52.9)	9(12.7)	5(23.8)	5(14.3)	1(4.3)	2(9.5)
	YES	165(80.5)	16(47.1)	62(87.3)	16(76.2)	30(85.7)	22(95.7)	19(90.5)

H&NC: head and neck cancer; CRC: colorectal cancer; ESOC: esophageal cancer; GC: gastric cancer; NSCLC: non small cell lung cancer; n: number of patients.

ADC: adenocarcinoma; Non-ADC: squamous cell carcinoma or adeno-squamous carcinoma; RT: radiotherapy; CT: chemotherapy; CRT: chemoradiotherapy.

### Safety profile

Treatment was well tolerated. The majority of the adverse events (AEs) were classified as mild or moderate (in spite of the causal relationship). No serious AE (SAE) occurred. Although the co-administered CT regimens were different for different indications, the safety profile of nimotuzumab was similar to previously reported studies [37–40]. Table 3 summarizes the System Organ Classification (SOC) of AEs by indication; gastrointestinal disorders (43%) such as nausea, diarrhea, and vomit were the most frequent AEs, followed by some investigations (38.3%) such as white blood cell decreased, neutrophil count decreased and platelet count decreased. Table 4 summarizes the Grade of AEs (381 events) classified by indication. Only 16.8% and 5.2% of patients had AEs in grade 3 and 4 respectively. Supplementary information demonstrates the SOC and Grade of all AEs in different indications. The Grade of all AEs in different nimotuzumab doses (mg/week) are presented in Table 5.

**Table 3:** System Organ Classification of adverse events

System Organ Class	NSCLC	ESOC	CRC	H&NC	GC	Others	Total (%)
	No. (%)	No. (%)	No. (%)	No. (%)	No. (%)	No. (%)	
Metabolism and nutrition disorders	1(2.3)	2(4.7)	3(2.8)	3(5.2)	2(2.3)	1(2.3)	12(31.5)
Musculoskeletal and connective tissue disorders	0	0	0	1(1.7)	1(1.1)	0	2(0.5)
Skin and subcutaneous tissue disorders	3(7.0)	1(2.3)	5(4.7)	2(3.4)	1(1.1)	2(4.5)	14(3.7)
General disorders and administration site conditions	2(4.7)	2(4.7)	3(2.8)	2(3.4)	6(6.9)	4(9.1)	19(5.0)
Nervous system disorders	0	1(2.3)	6(5.7)	3(5.2)	2(2.3)	5(11.4)	17(4.5)
Gastrointestinal disorders	19(44.2)	20(46.5)	53(50.0)	20(34.5)	38(43.7)	14(31.8)	164(43.0)
Vascular disorders	0	0	0	0	1(1.1)	0	1(0.3)
Blood and lymphatic system disorders	0	0	2(1.9)	1(1.7)	3(3.4)	0	6(1.6)
Investigations	18(41.9)	17(39.5)	34(32.1)	26(44.8)	33(37.9)	18(40.9)	146(38.3)
Total (%)	43(11.3)	43(11.3)	106(27.8)	58(15.2)	87(22.8)	44(11.5)	381(100)

NSCLC: non small cell lung cancer; ESOC: esophageal cancer; H&NC: head and neck cancer; CRC: colorectal cancer; GC: gastric cancer.

No. (%): times and percent of AE occurred in patients of each indication; Total (%): total number of AE times in each indication or each system organ

**Table 4:** Grade of adverse events classified by indications

Indications	Grade 1		Grade 2		Grade 3		Grade 4	
	N	%	N	%	N	%	N	%
NSCLC	13	8.2%	23	16.5%	5	7.8%	2	10.0%
ESOC	19	12.0%	11	7.9%	10	15.6%	3	15.0%
CRC	41	25.9%	43	30.9%	18	28.1%	4	20.0%
H&NC	23	14.6%	19	13.7%	12	18.8%	4	20.0%
GC	47	29.7%	28	20.1%	10	15.6%	2	10.0%
Others	15	9.5%	15	10.8%	9	14.1%	5	25.0%
Total	158	100.0%	139	100.0%	64	100.0%	20	100.0%
Indication/Total	158/381	**41.5**	139/381	**36.5**	64/381	**16.8**	20/381	**5.2**

NSCLC: non small cell lung cancer; ESOC: esophageal cancer; H&NC: head and neck cancer; CRC: colorectal cancer; GC: gastric cancer

**Table 5:** Grade of adverse events classified by nimotuzumab doses

Doses nimotuzumab (mg/w*)	100 (n=6)		200 (n=47)		250 (n=1)		300 (n=12)		400 (n=130)		500 (n=1)		600 (n=8)		Total (n=205)	
Grade of AE	n	Times	n	Times	n	Times	n	Times	n	Times	n	Times	n	Times	n	Times
Patients with AE	6	24	42	94	0	0	9	19	113	219	1	1	8	24	179	381
Patients with No-AE	0	0	5	0	1	0	3	0	17	0	0	0	0	0	26	0
Grade 1	5	8	23	33	0	0	6	8	59	95	0	0	7	14	100	158
Grade 2	5	10	18	33	0	0	5	5	61	84	1	1	5	6	95	139
Grade 3	*1*	*4*	*19*	*26*	*0*	*0*	*4*	*6*	*20*	*26*	*0*	*0*	*2*	*2*	*46*	*64*
Grade 4	*1*	*1*	*1*	*3*	*0*	*0*	*0*	*0*	*13*	*14*	*0*	*0*	*1*	*2*	*16*	*20*
Grade 5	*0*	*0*	*0*	*0*	*0*	*0*	*0*	*0*	*0*	*0*	*0*	*0*	*0*	*0*	*0*	*0*
p value (compare with a total)	*0.35*		*0.73*		*0.01*		*0.25*		*0.93*		*0.70*		*0.29*		*1.00*	

NSCLC: non small cell lung cancer; ESOC: esophageal cancer; H&NC: head and neck cancer; CRC: colorectal cancer; GC: gastric cancer

n: number of patients in each dosage group; AE: adverse event, if one patient occurred multiple AE in different grade, it was recorded individually. The severity of AE was classified by NCI-CTCAE 4.03.

* mg / w: milligrams of antibody (fix doses) administer per week.

### Antitumor response

A total of 171 patients (83.4%) were evaluated by RECISIT criteria at least one time after the treatment of nimotuzumab. The antitumor response was reported as objective response rate (ORR, complete response (CR) + partial response (PR)) and disease control rate (DCR, CR+ PR+ stable disease (SD)). The antitumor responses in different indications are shown in Table 6. According to the ORR, H&NC patients were the best responders (55.9%), followed by ESOC patients and those who with CRC (42.9% and 28.2% respectively). In regards to the DCR, all patients reached more than 50%, H&NC was the most efficient (91.2%), followed by NSCLC (78.3%) and CRC (67.6%).

**Table 6:** Antitumor Response Profile by Pearson Chi-square analysis

Factors		n	TOTAL (n=205)		H&NC (n=34)		GC (n=35)	
			N(ORR)%	N(DCR)%	N(ORR)%	N(DCR)%	N(ORR)%	N(DCR)%
Total		205	66(32.2)	139(67.8)	19(55.9)	31(91.2)	8(22.9)	19(54.3)
Sex	Male	139	48(34.5)	102(73.4)	15(57.7)	26(100.0)	6(25.0)	14(58.3)
	Female	66	18(27.3)	37(56.1)	4(50.0)	5(62.5)	2(18.2)	5(45.5)
	p*		*0.445*	***0.002***	*0.187*	***0.005***	*0.193*	***0.028***
Age	<60y	137	44(32.1)	92(67.2)	12(52.2)	23(100.0)	7(30.4)	13(56.5)
	≥60y	68	22(32.4)	47(69.1)	7(63.6)	8(72.7)	1(8.3)	6(50.0)
	p*		*0.515*	*0.240*	*0.220*	***0.032***	*0.285*	*0.863*
Histology	ADC	140	39(27.9)	90(64.3)	2 (100.0)	2(100.0)	8 (24.2)	18(54.6)
	non-ADC	65	27(41.5)	49(75.4)	17 (53.1)	29(90.6)	0(0)	1(50.0)
	p*		*0.128*	*0.283*	*0.432*	*0.902*	*0.367*	*0.471*
Treatment lines	1st	40	20(50.0)	30(75.0)	10(55.6)	16(88.9)	3(60.0)	3(60.0)
	2nd	81	26(32.1)	57(70.4)	4(44.4)	8(88.9)	4(19.0)	13(61.9)
	3rd	41	13(31.7)	25(61.0)	3(100.0)	3(100.0)	1(12.5)	3(37.5)
	>3rd	43	7(16.3)	27(62.8)	2(50.0)	4(100.0)	0(0)	0(0)
	p*		***0.036***	*0.702*	*0.683*	*0.937*	*0.208*	*0.345*
Nimotuzumab frequency	≤6	73	17(23.3)	39(53.4)	6 (54.6)	9(81.8)	0(0)	3(23.1)
	>6	132	49(37.1)	100(75.8)	13 (56.5)	22(95.7)	8(36.4)	16(72.7)
	p*		***0.050***	***0.004***	*0.85*	*0.335*	***0.004***	***0.006***
Nimotuzumab doses (mg/w)	≤200	54	20(37.0)	36(66.7)	7 (53.9)	11(84.6)	1 (16.7)	4(66.7)
	>200	151	46(30.5)	103(68.2)	12 (57.1)	20(95.2)	7 (24.1)	15(51.7)
	p*		*0.567*	*0.436*	*0.434*	*0.400*	*0.181*	*0.258*

n: number of patients who have received at least one evaluation. p: statistical p value.

Bold text indicates statistically significant P-values (<0.05)

* compared between two groups of each factor.

The correlation between different factors (sex, age, histology, previous treatment, treatment lines, doses and frequency of nimotuzumab) and antitumor response were investigated. The difference in DCR between male and female was evident in all patients, with more benefit for men than women had (73.4% vs 56.1%, p=0.002), similar results were found in H&NC and GC (p=0.005 and 0.028, respectively), but no difference was found in NSCLC, ESOC and CRC patients (Data not shown). No obvious difference of ORR was found between men and women in total population or in different tumor types. According to the age, only the DCR of patients with H&NC showed significant difference (p=0.032, 100% vs 72.7% in age <60 years vs ≥ 60 years). Concerning the histology, no significant difference was observed between patients with ADC and non-ADC. The therapeutic schedules (nimotuzumab+ CT) were used as 1st, 2nd, 3rd, and >3rd lines treatment in 40, 81, 41, and 43 patients, and the ORR of the four groups was 50.0%, 32.1%, 31.7%, and 16.3% respectively, with significant difference among groups (P=0.036). However, no difference of treatment lines was found in DCR among groups (p=0.702). The patients received more than six doses have significant improvement on the ORR (23.3% vs. 37.1%, p=0.050) and DCR in total (75.8% vs. 53.4%, p=0.004) and GC (72.7% vs. 23.1%, p=0.006), and ORR in GC (36.4% vs. 0%, p=0.004). In addition, no significant difference was found between previous treatment (surgery, RT/CRT, CT) and antitumor response (data not shown).

### Survival

Univariate analysis was performed to determinate the association between factors (sex, age, histology, differentiation, surgery history, RT/CRT history, CT history, treatment lines, nimotuzumab >200mg/week and nimotuzumab >6 doses) and overall survival (OS) or progression free survival (PFS). The analysis for OS showed that the statistically significant variables were age>60 years in H&NC with the hazard ratio (HR) of 4.65(1.29-16.74) (p=0.019), and nimotuzumab > 200mg/week and nimotuzumab >6 doses in GC with the HR of 0.08(0.02-0.39) and 0.21(0.05-0.98) (p=0.002 and 0.047, respectively). Univariate analysis for PFS showed that surgery history and nimotuzumab >6 doses were significantly associated with CRC patients' PFS with the HR of 0.43(0.20-0.93) and 0.39(0.17-0.85) (p=0.037 and 0.020, respectively); male gender was statistically significant related factor for PFS in GC patients with the HR of 3.69(1.08-12.64) (p=0.038). The other clinical and treatment parameters have no correlation with OS or PFS in different tumor types (Data not shown). Further, only those variables found to be significantly associated with outcome in univariate analyses were included in multivariable analyses.

As shown in Table 7A, prior surgery and nimotuzumab >6 doses were identified as independent predictive factors for increased PFS in CRC with an HR of 0.44 (95% CI: 0.20-0.95, p=0.037) and HR of 0.40 (95% CI: 0.18-0.88, p=0.020). A similar pattern can be seen in GC. Male and nimotuzumab > 6 doses were independent predictive factors for increased PFS with HR 0.17 (95% CI: 0.04-0.73, p=0.020) and 0.15 (95% CI: 0.02-0.98, p=0.048). In Table 7B, younger age was a significant independent predictive factor for improved OS of H&NC patients, with HR 0.28 (95% CI: 0.07-1.19, p=0.019). Nimotuzumab >200mg/week was a significant factor related with increased OS in GC, with a HR of 0.11 (95% CI: 0.02-0.59, p=0.01).

**Table 7:** Factors Related with OS and PFS in Multivariate Cox Regression analysis

**A: Multivariate analysis for PFS**			
**Tumor types**	**Variable**	**HR (95% CI)**	**p**
CRC	Surgery history	0.44(0.20-0.95)	**0.037**
	nimo>6 doses	0.40(0.18-0.88)	**0.020**
GC	Male	0.17(0.04-0.73)	**0.020**
	nimo>6 doses	0.15(0.02-0.98)	**0.048**
**B: Multivariate analysis for OS**			
**Tumor types**	**Variable**	**HR (95% CI)**	**p**
GC	nimo>200mg/week	0.11(0.02-0.59)	**0.010**
H&NC	Age>60 years	0.28(0.07-1.19)	**0.019**

PFS: progression free survival, OS: overall survival

**Figure 1:**
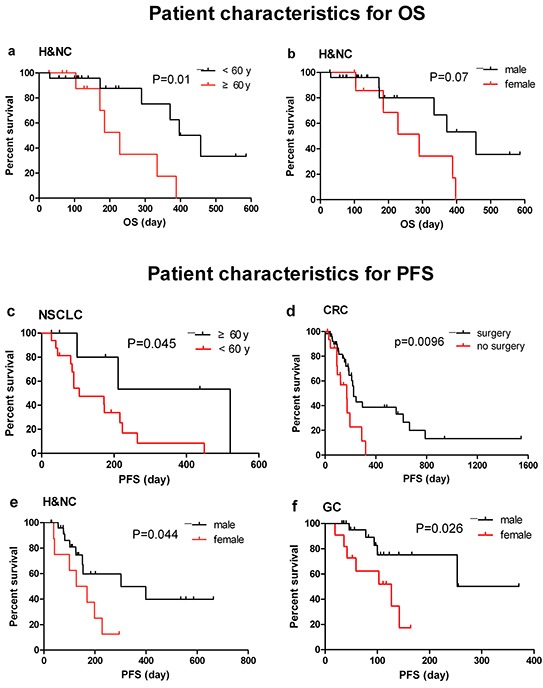
Potential patient characteristics related with OS and PFS in 5 indications **a.** OS of H&NC patients by age; **b.** OS of H&NC patients by sex; **c.** PFS of NSCLC patients by age; **d.** PFS of CRC patients by prior surgery history; **e.** PFS of H&NC patients by sex; **f.** PFS of GC patients by sex.

**Figure 2:**
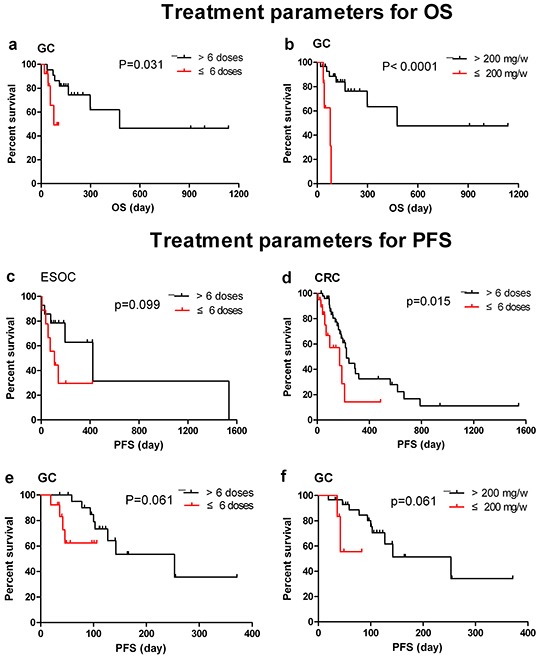
Potential treatment parameters related with for OS and PFS in 5 indications **a.** OS of GC patients treated by nimotuzumab > or ≤6 doses; **b.** OS of GC patients treated by nimotuzumab > or ≤ 200mg/week; **c.** PFS of ESOC patients treated by nimotuzumab > or ≤6 doses; **d.** PFS of CRC patients treated by nimotuzumab > or ≤ 200mg/week; **e.** PFS of GC patients treated by nimotuzumab > or ≤6 doses; **f.** PFS of GC patients treated by nimotuzumab > or ≤ 200mg/week.

Table 8 and 9 reports the correlation between different factors (sex, age, surgical history, dose and frequency of nimotuzumab) and survival. Age, dose and duration of therapy were also considered to correlate with OS (Table 8). Sex, age, dose and previous treatment (surgery and RT) were related with PFS (Table 9). As shown in Figure 1 and 2, sex, previous treatment, and nimotuzumab > 6 doses and > 200 mg/week might be predictive factors for the likelihood of benefit from nimotuzumab therapy.

**Table 8:** Overall Survival profile by Log-Rank analysis

Factors		OS																			
		NSCLC				ESOC				CRC				H&NC				GC			
		OS (day)	95% CI		p	OS (day)	95% CI		p	OS (day)	95% CI		p	OS (day)	95% CI		p	OS (day)	95% CI		p
General OS		417	209	624.7		420	171	669		655	314	996		371	261	481		476	86	866	
Sex	Male	549	85.6	1012	0.93	420	171	669	NA	1393	367	2419	0.38	457	314	600	0.07	476	28.9	923	0.54
	Female	417	.	.		NA	NA	NA		525	291	759		290	173	407		298	0	703	
Age	<60y	351	201	501.4	0.58	.	.	.	0.48	867	274	1460	0.83	457	354	560	**0.01**	476	0	1021	0.73
	≥60y	549	4.11	1094		270	18.4	522		643	481	805		228	166	290		298	0	612	
Surgery	YES	417			0.197	420	181.7	658.3	0.832	525	293.7	756.7	0.21	388	280.9	495.1	0.42	476	139.3	812.7	0.44
	NO	278	48.4	507.6		.	.	.		867	474.4	756.7		228	20.8	435.2		166	.	.	
Nimotuzumab frequency	≤6	417	104	729.9	0.22	270	191	349	0.12	391	341	441	0.32	228	20.9	435	0.37	77	.	.	**0.03**
	>6	549	168	930.3		.	.	.		655	317	993		388	341	435		476	.	.	
Nimotuzumab doses (mg/w)	≤200	278	9.14	546.9	0.99	270	.	.	0.67	270	.	.	0.94	334	67.7	600	0.93	77	23.1	131	**0.00**
	>200	417	254	579.7		420	180	660		420	180	660		388	280	496		476	.	.	

Bold text indicates statistically significant (P-values <0.05)

**Table 9:** Progression Free Survival profile by Log-Rank analysis

Factors		PFS																			
		NSCLC				ESOC				CRC				H&NC				GC			
		PFS (day)	95% CI		p	PFS (day)	95% CI		p	PFS (day)	95% CI		p	PFS (day)	95% CI		p	PFS (day)	95% CI		p
General PFS		173	33.1	312.9		196	0	411		217	181	253		198	102	294		142	18.8	265	
Sex	Male	173	112	233.9	0.23	196	0	411	NA	210	170	250	0.76	302	0	635	**0.04**	.	.	.	**0.03**
	Female	89	76.2	101.8		NA	NA	NA		225	114	336		127	31.4	223		127	42.4	212	
Age	<60y	104	21.9	186.1	0.04	196	0	408	0.56	210	78.2	342	0.59	198	0	415	0.48	253	0	517	0.62
	≥60y	521								225	171	279		151	61.6	240		127	12.5	242	
Surgery	NO	98	0	227.6	0.34	1536			0.10	171	107	235	0.03	169	96	242	0.62	142	106	178	0.80
	YES	211	80.6	341.4		109	0	244		225	188	262		228	0	466		253	30.3	476	
Nimotuzumab frequency	≤6	89	40.5	137.5	0.27	109	28.2	190	0.07	171	4.11	338	0.01	228	72.7	383	0.85	.	.	.	0.06
	>6	211	146	275.8		423	83.2	763		225	185	265		198	36.8	359		253	103	403	
Nimotuzumab doses (mg/w)	≤200	264	81.5	446.5	0.55	141			0.58	163	75.5	251	0.45	399	85.4	713	0.30	.	.	.	0.06
	>200	173	40.1	305.9		423	80.4	766	217		174	260		169	105	233		253	103	403	

Bold text indicates statistically significant (P-values <0.05)

**Figure 3:**
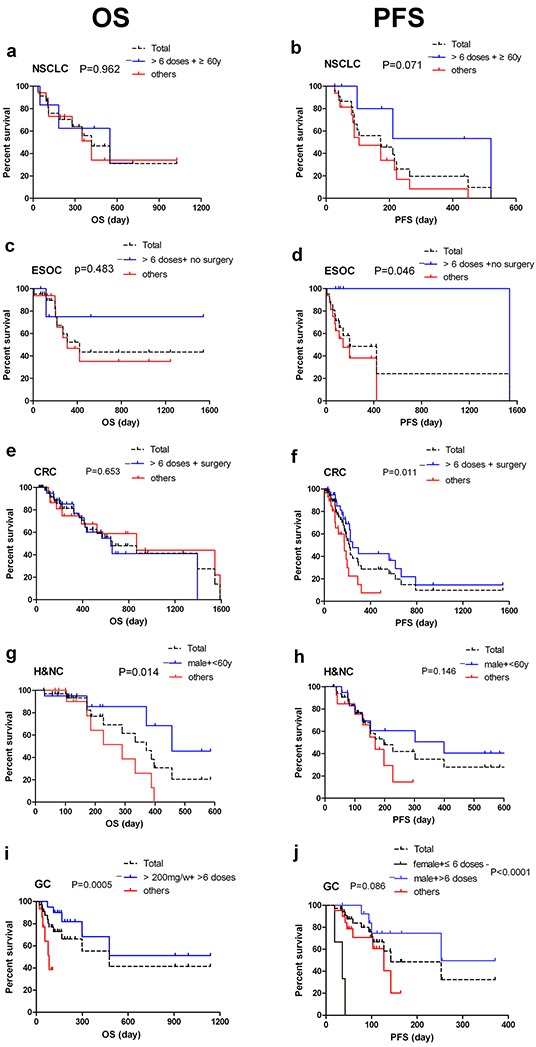
Factors to predict patient prognosis and response to nimotuzumab therapy in 5 indications **a. OS of NSCLC** by nimotuzumab doses and age. Median OS for (>6 doses+ ≥60y) group and others group was 549 days vs 417 days (n=7 vs 16), with HR 0.37 (95% CI 0.10 to 1.16); **b. PFS of NSCLC** by nimotuzumab doses and age. Median PFS for (>6 doses+ ≥60y) group and others group was 521 days vs 104 days (n=7 vs 16), with HR 0.40 (95% CI 0.15 to 1.10); **c. OS of ESOC** by frequency of nimotuzumab and prior surgery history. Median OS for (>6 doses+ no surgery) group and others group was undefined vs 308 days (n=5 vs 16), with HR 0.48 (95% CI 0.11 to 2.88); **d. PFS of ESOC** by frequency of nimotuzumab and prior surgery history. Median PFS for (>6 doses+ no surgery) group and others group was 1536 days vs 141 days (n=5 vs 16), with HR 0.19 (95% CI 0.07 to 0.83); **e. OS of CRC** by frequency of nimotuzumab and prior surgery history. Median OS for (>6 doses+ surgery) group and others group was 643 vs 867 days (n=42 vs 29), with HR 1.19 (95% CI 0.55 to 2.67); **f. PFS of CRC** by frequency of nimotuzumab and prior surgery history. Median PFS for (>6 doses+ surgery) group and others group was 244 days vs 173 days (n=42 vs 29), with HR 0.44 (95% CI 0.17 to 0.77). **g. OS of H&NC** by sex and age. Median OS for (male + <60y) group and others group was 457 vs 290 days (n=20 vs 14), with HR 0.26 (95% CI 0.06 to 0.70); **h. PFS of H&NC** by sex and age. Median PFS for (male + <60y) group and others group was 399 days vs 169 days (n=20 vs 14), with HR 0.51 (95% CI 0.16 to 1.26); **i. OS of GC** by doses and frequency of nimotuzumab. Median OS for (>200mg/w+ >6 doses) group and others group was undefined vs 83 days (n=20 vs 15), with HR 0.18 (95% CI 0.01 to 0.23); **j. PFS of GC** by sex and frequency of nimotuzumab. Median PFS for (male + >6 doses) group and others was 253 days vs 127 days (n=14 vs 21), and median PFS of (female+≤6 doses) group was only 36 days (n=3), with HR 0.08 (95% CI 1.031e-005 to 0.008).

The impact of these factors on survival in 5 clinical indications was further illustrated in Figure 3. In the NSCLC arm, there was a trend for longer PFS in patients with age ≥ 60 years and had received more than six doses of nimotuzumab (p=0.071, Figure 3b). In the ESOC arm, the patients without prior surgery had longer PFS when they received more than six doses of nimotuzumab (p=0.046, Figure 3d). In the CRC arm, a significant difference was also observed in PFS when patients had prior surgery and received nimotuzumab over six doses as maintenance treatments (p=0.011, Figure 3f). In the H&NC arm, males with age < 60 years survived longer than others did (p=0.014, Figure 3g). In the GC arm, patients who received more than 200 mg/week and six doses had longer OS than the others (p=0.0005, Figure 3i), and males who received nimotuzumab over six doses had obvious increase in PFS than females who only received nimotuzumab in less than six doses (p<0.0001, Figure 3j).

## DISCUSSION

Nimotuzumab has shown excellent antitumor activity when combined with RT or CRT in advanced H&NC [21], ESOC [30] and glioma [41] patients. In recent years, some clinical trials in China have been launched to test the clinical benefit of nimotuzumab combined with CT in different types of cancer: ESOC [42–44], NPC [45], GC [46], pancreatic cancer [47], glioma [48] and NSCLC [39, 49, 50]. Ideally, the efficacy of EGFR target agents should be related with some molecular factors. Several biomarkers such as EGFR gene copy number, KRAS mutation, AKT, ERK has been investigated extensively, but results obtained still remain controversial, suggesting potential off-target effects and yet-discovered molecular co-factors [8, 42, 51, 52]. In absence of correlative molecular data, however, oncologists found that some clinical characteristics also could predict the effect of EGFR inhibitors. Examples include patient sex, histology, and smoking history that were found to be associated with the clinical benefit of erlotinib [53]. Our retrospective study was designed to assess the safety and efficacy of nimotuzumab in combination with chemotherapy in advanced tumors from epithelial origin in Chinese patients. The clinical characteristics (age, sex, histology, previous treatment, tumor differentiation, tumor stage) and treatment predictive factors (dose, frequency and treatment lines) were also analyzed.

In this report, treatment with nimotuzumab in combination with CT was well tolerated. No SAE was attributed to nimotuzumab in combination with CT. A total of 381 AEs were collected. In general, the majority of AEs were gastrointestinal disorders (43.0%), laboratory investigations (38.3%), and metabolism and nutrition disorders (31.5%) (Table 3). Skin and subcutaneous tissue disorders only accounted 3.7 % of the AEs. Only 22% AEs were reported as grade 3 or 4 (Table 4).

The addition of nimotuzumab to different chemotherapeutic regimens for different types of tumors did not increase the toxicity of CT. In contrast, SAEs have been reported for other widely used anti-EGFR antibodies already in the market, such as cetuximab and panitumumab. Cetuximab can induce severe acneiform rash in 1-17% patients, severe infusion reactions in approximately 3% of patients as well as cardiopulmonary arrest and sudden death in up to 2% of the patients [54]. Panitumumab engenders dermatologic toxicities in 89% of patients (12% severe) and severe infusion reactions in approximately 1% of patients [55]. In our set of patients, skin and subcutaneous tissue disorders just accounted for 2.6% (10/381) of all the AEs in grade 1 and 2. Studies of nimotuzumab in various tumors also demonstrated remarkable dermatological safety [40, 56]. This favorable toxicity profile can be explained by a kinetic binding model of anti-EGFR antibodies, where intermediate affinity of nimotuzumab (KD = 10^−9^M) to the receptor, results in a high tumor uptake and low uptake into normal tissues [20, 21].

The excellent safety profile of nimotuzumab allows its long-term use and provides a better quality of life of patients [17]. Over 60% patients received nimotuzuamb over six administrations as maintenance therapy (the maximum number was 60 times) in our study. Patients who received more than 100 mg (200-600 mg) weekly showed a similar safety profile with patients in fewer dosages. No significant difference was found between doses related with the total of AE (Table 5).

Patients with CRC, GC, H&NC, NSCLC and ESOC were selected for efficacy analysis; each indication had more than 20 patients. Most of the patients were in Stage IV (97.6%), 92.2% patients had prior treatment, and 80.5% patients had previously received chemotherapy (>40% patients received at least two regimens before). Surprisingly, it was found that the ORR and DCR arrived 31.7% and 67.3% in total, which makes us believe this could be an alternative therapy even after tumor progression by 1^st^-3^rd^ lines treatment and possibly beyond.

With regard to the antitumor response, analysis by some risk factors showed that men had better responses than women did (DCR: 73.4% vs 56.1%, p=0.002). Similarly, H&NC patients younger than 60 years had 100% DCR, compared to 72.7% observed in their older counterparts (p=0.032). The therapeutic schedules (nimotuzumab+ CT) that used as 1st line treatment had better response than those treated as 2nd, 3rd and >3rd lines treatment (50.0%, 32.1%, 31.7%, and 16.3% respectively).

Consequently, we performed the univariate and multivariate analysis and Log-Rank test by clinical indications to identify some special sub-populations that may better benefit from the nimotuzumab therapy.

For 34 H&NC patients, 90% in Stage IV and 85% were squamous cell carcinoma. There were 14 cases of NPC, 4 cases of hypopharyngeal cancer, 4 cases of oral cancer, 3 cases tongue cancer, 2 cases of laryngeal cancer, 2 cases of maxillary sinus carcinoma, 1 case parotid gland one, 1 case of lip cancer, and 1 case orbital cancer were included. Sixteen (47.1%) patients received multi-line chemotherapy (including platinum-based regimen, data not shown). Since 2014, the NCCN Guidelines recommended for recurrent, unresectable or metastatic disease in H&NC using different regimens of CT including cisplatin/docetaxel/cetuximab[57], cisplatin/ paclitaxel/cetuximab [58] and cisplatin/gencitabine for non NPC cancer [45]. The ORR of our study was 55.9%, DCR was 91.2%, median PFS was 6.4 months and the median OS 12.4 months, 1-year OS rate was 53.8% (Table 6–8). These results were better than the previous results from CT alone or cetuximab combined with CT regimens in recurrent or metastatic head and neck tumors, with ORR (20% vs 36%, P < 0.001), median PFS (3.3 vs. 5.6 months, P < 0.001) and OS (7.4 vs 10.1 months, P = 0.04 [59]. Multivariate analysis showed that age was a significant predictive factor for the patient's survival (Table 7). Males with age < 60 years survived significant longer than others did (457 days vs 290 days, p=0.014, Figure 3g). Further, patients with NPC in our report showed an ORR in 50% and the 100% of DCR, the median PFS was 6.5 months, 1 year PFS rate was 44.6%, the median OS 12.9 months, 1 year OS at a rate of 66.7% (data not shown). Similar results were previously reported by Chinese oncologists in endemic NPC areas that nimotuzumab in combination with gemcitabine in patients with advanced metastatic NPC obtained an ORR in 61.5% [60] and a combination of nimotuzumab with paclitaxel and cisplatin in the treatment of recurrent NPC had the ORR in 64.28% and DCR in 92.85% [61]. Until this report, no other result was published about the treatment of nimotuzumab combined with CT in advanced H&NC.

Esophageal cancer is one of the most common malignant tumors worldwide and its incidence is increasing. In western countries, esophageal adenocarcinoma in esophagogastric junction cancer has dramatically increased in incidence and now accounts for 60 ~ 70% of esophageal cancer [62], but in China, squamous carcinoma is still the main pathological subtype, accounting for more than 90% [63]. Currently, there is no consensus treatment that is recommended for ESOC. Several phase III trials have not found significant differences in outcome between various CT regimens, such as Irinotecan / 5-FU vs cisplatin / 5FU (PFS 6 vs. 9 months and OS 9 vs. 8.7 months) [64], FOLFIRI vs ECX (Epirubicin/ oxaliplatin/ 5-FU) (OS 9.5 vs. 9.7 months and PFS 5.3 vs. 5.8 months) [65], FLO vs FPL (PFS 5.8 vs. 3.9 months, OS 10.8 vs. 8.8 months) [66]. The median OS remains in 8.7-10.8 months and PFS in 3.9-9 months. All of the 21 ESOC patients analyzed in this retrospective study were male, had squamous cell carcinoma, and were in Stage IV. The median OS was 14 months (420 days), PFS was 6.5 months (196 days), ORR was 42.9%, and DCR was 61.9%, respectively. Our results were similar to a previous study conducted in China, which enrolled 19 patients in advanced stage, treated by nimotuzumab in combination with cisplatin/5FU, the ORR and DCR was 42.1% and 68.4%, respectively [44]. In contrast, cetuximab in combination with cisplatin/5-FU as the first-line therapy for metastatic ESOC had an OS of 9.5 months and DCR in 75% [27]. When Cetuximab combined with irinotecan as the second-line treatment for platinum-resistant gastroesophageal adenocarcinoma, six cases of PR (11%), SD of 37%, median PFS 2.8 months, and OS 6.1 months were obtained [29]. In our study, all the patients had Stage IV disease and 76.2% patients had received prior platinum-based chemotherapy, the efficacy was superior to that of cetuximab. Patients without prior surgery who used more than six doses of nimotuzumab, had a longer PFS than other groups (p=0.046, Figure 3d).

Gastric cancer is the third leading cause of death by cancer in China [67]. Several combination regimens have been used as first-line treatment, including DCF (docetaxel/cisplatin/5-FU) [68], ECF (Epirubicin/cisplatin/5-FU) [69], ECF modification [70], cisplatin/capecitabine [71] and cisplatin/5-FU [66, 72]. However, the median survival has not exceeded 8–13 months [73, 74]. A phase II controlled clinical trial using nimotuzumab in combination with irinotecan as a second-line therapy in advanced gastric cancer patients (83 patients: 40 in nimotuzumab group versus 43 in control group) reported that median PFS was 73.0 versus 85.0 days (P = 0.5668), median OS and RR at 18 months were 250.5 versus 232.0 days (P = 0.9778) and 18.4 versus 10.3%, respectively [37]. With a similar sample size in the present analysis, 35 patients with advanced gastric cancer were treated with nimotuzumab in combination with CT, reached a OS of 15.86 months (476 days) and a PFS of 4.73months (142 days), ORR 22.9% and DCR 54.3%. Furthermore, Patients received more than 200 mg/week in at least six doses had longer OS than the others (p=0.0005), and males who received nimotuzumab >6 doses have significant increase in PFS than females receiving only ≤6 doses of nimotuzumab (p<0.0001). Our results support the possible use of the nimotuzumab in gastric cancer [37].

Twenty-three Patients with advanced NSCLC were analyzed. 91.3% patients were Stage IV and 60.9% were ADC. 22 cases (95.7%) received prior CT and 20 cases (90.9%) received at least 2 CT regimens. In the 22 cases, only 4 patients (18.2%) did not receive targeted therapy. In those cases, the ORR was 13.0%, which was lower than previous reports (25%-35%). However, the DCR was 78.3 %, the OS reached 13.9 months and the PFS was 5.76 months, which were similar to the data has been published [75]. Univariate and multivariate analysis have not found any predictive factors correlated with OS and PFS. The patients older than 60 years of age and treated with nimotuzumab over six doses, had a longer PFS than the others, but this was not a statistically significant difference (521 days vs 104 days, P=0.07). In NSCLC, the relationship between age and survival is inverse with a trend towards statistical significance in univariate analysis. The outcomes of young and old patients with lung cancer have been previously studied, but the results are inconsistent [76, 77]. Most studies compared only the outcomes of younger and older patients, but not the outcomes of different age groups. Our study showed that the median PFS of patients aged ≤ 60 years was significantly shorter than older patients with NSCLC (Table 8 and 9). No significant difference was found in OS. A similar result was obtained in a recent Chinese study[78]. The explanation for the survival difference remains unclear.

Cetuximab has been approval by the Food and Drug Administration (FDA) to treat patients with CRC in combination with FOLFIRI for first-line treatment, in combination with irinotecan in patients who are refractory to irinotecan-based chemotherapy, or as a single agent in patients who have failed oxaliplatin-and irinotecan-based chemotherapy or who are intolerant to irinotecan [54]. Use of cetuximab in combination with FOLFIRI don't reach significant difference concerning with OS but for PFS 8.9 vs. 8.1 months (p=0.036) [54]. In patients with metastatic colorectal cancer treated with CT or best supportive care (BSC), the OS was 6.1 vs. 4.6 months, but when the KARS mutation is taken in account, there is an OS increase for a wide-type KRAS patients, 8.6 vs. 5.0 months. The set of patients analyzed in this report (n=71) were all in Stage IV. 39 patients had a known KRAS gene status and of these, 37 patients (94.9%) were wild-type KRAS. 63 cases (87.5%) had received prior chemotherapy, in which over 50% patients received at least 2 CT regimens. 13 cases received prior immunotherapy with bevacizumab or cetuximab. The general OS for those patients was 21.8 months (655days) and PFS was 7.2 months (217 days), which were significantly longer than previous reports. Multivariate analysis indicated that prior surgery and nimotuzumab> 6 doses were related with PFS significantly. The patients who had prior surgery and received more than six doses of nimotuzumab have better PFS than others (p=0.011). The efficacy of nimotuzumab combined with CT in the treatment of advanced colorectal cancer was slightly better than that of cetuximab, and with mild adverse reactions. This finding warrants further investigation.

It was found that age, patient sex and previous surgery could be factors to consider in antitumor response. Prior surgery in CRC has a positive correlation with PFS (p=0.037) and in ESOC this factor had a negative influence (p=0.012). This factor has no influence on other indications. Patients younger than 60 years appear to have a greater survival benefit to nimotuzumab treatment of H&NC. Nimotuzumab > 6 doses are related with longer PFS in CRC and GC, and Nimotuzumab > 200mg/weekly is related with longer OS in GC. Even in those patients refractory to combination treatment (n=33), the PFS of patients got >6 doses (n=16) was significantly longer than those only received ≤ 6 doses (n=17), with the median PFS of 92±7.9 days vs 41±0.8 days (p=0.002) and the median OS of 655±348.9 days vs 198±67.4 days (p=0.12, data not shown). For H&NC, statistical significance was found to benefit males less than 60 years, while in GC it was found that patients receiving the highest number of doses over 200 mg had a longer survival.

In conclusion, nimotuzumab administered weekly was well tolerated up to 600 mg in Chinese patients. Our results support that >200 mg weekly and more than six in frequency (maintenance therapy) can be the dosing schedule recommended for further clinical studies, especially for the combination of nimotuzumab with chemotherapy. Additionally, it could be possible for combination treatment of nimotuzumab and another targeted therapy or immunomodulatory checkpoint antibody based on its safety profile.

## MATERIALS AND METHODS

### Patients

Patients with non-resectable, advanced epithelial malignant tumors treated with nimotuzuamb combined with chemotherapy at Cancer Hospital, Chinese Academy of Medical Sciences (CAMS) between May 1, 2010 and August 1, 2015 were indentified. The patients with unknown metastases and poor performance status (ECOG)>2 were not selected. Clinical data of patients was collected, including diagnosis, age, gender, pathological type, tumor stage, tumor grade, pretreatment history (surgery, RT/CRT, CT), recurrence or metastasis time after surgery, metastasis site, history of nimotuzumab (delivery time, dosage, dosing frequency, combined chemotherapy regimens). The retrospective study was conducted in accordance with the Declaration of Helsinki and the International Conference of Harmonization Good Clinical Practices (ICH-GCP).

### Treatment

All of the patients received treatment with nimotuzumab in combination with chemotherapy. The antibody was administered by intravenous injection, in 250 mL of saline solution. Doses between 100-600 mg (fixed dose) were administered weekly, in combination with different schedules of chemotherapy, which was depending on the classification of tumors and the corresponding Chinese Guideline recommendations). Due to the safety profile previously reported for this antibody, the clinicians used maintenance therapy (>6 doses with the original dose) in 64.4% (132/205) of patients.

### Evaluation

The clinical endpoint of interest was safety, ORR, DCR, PFS and OS in each indication. The information was collected from the clinical historical record of individual patients. All the AEs were collected and graded by National Cancer Institute's Common Toxicity Criteria (NCI CTC) version 4.03. The antitumor response was evaluated according to Response Evaluation Criteria in Solid Tumors (RECIST), version 1.0 and 1.1 guidelines [79, 80]. ORR was calculated as CR+PR, DCR was calculated as CR+PR+SD. OS was defined as the date of the first nimotuzumab/chemotherapy infusion to the date of death or last contact (visit and telephone). PFS was defined as the time from the first injection of nimotuzumab to the date of progression or death.

### Statistical analysis

For each indication, the relationship between each variable with ORR or DCR was performed using Pearson Chi-square tests. The log rank test was used to analyze the association between each variable with OS or PFS, with its associated 95% confidence interval (95% CI). Cox's proportional hazard regression models were conducted for multivariate survival analyses. Proportional hazard assumption was evaluated by examining plots of residuals and by including time-dependent covariates in the models. *p*<0.05 represent significant differences. All the data were analyzed by SPSS software (version 18.0, IBM). GraphPad Prism (version 6.0, GraphPad Software) was used to draft the figure of Kaplan-Meier curve.

## SUPPLEMENTARY TABLE




