# Supplementary material for: Treatment outcome of nimotuzumab plus chemotherapy in advanced cancer patients: a single institute experience
Source: Oncotarget. 2016 Mar 31;7(22):33391–407. doi: 10.18632/oncotarget.8516 (PMC5078104; doi:10.18632/oncotarget.8516)
Supplement: Supplementary file 1 [file oncotarget-07-33391-s001.pdf]

Treatment outcome of nimotuzumab plus chemotherapy in advanced cancer patients: a single institute experience

SUPPLEMENTARY TABLE

Supplementary Table S1: Supplementary: System Organ classification and Grade of all adverse events in different tumor localizations.

See Supplementary File 1
