# Supplementary material for: Treatment outcome of nimotuzumab plus chemotherapy in advanced cancer patients: a single institute experience
Source: Oncotarget. 2016 Mar 31;7(22):33391–407. doi: 10.18632/oncotarget.8516 (PMC5078104; doi:10.18632/oncotarget.8516)
Supplement: Supplementary file 2 [file oncotarget-07-33391-s002.docx]

Table 1. Supplementary: System Organ classification and Grade of all adverse events in different tumor localizations

| System Organ Class | Adverse event | NSCLC  (Grade) | | | | ESOC  (Grade) | | | | CRC  (Grade) | | | | H&NC  (Grade) | | | | GC  (Grade) | | | | Others  (Grade) | | | | Total  (Grade) | | | |
| --- | --- | --- | --- | --- | --- | --- | --- | --- | --- | --- | --- | --- | --- | --- | --- | --- | --- | --- | --- | --- | --- | --- | --- | --- | --- | --- | --- | --- | --- |
|  |  | **1** | **2** | **3** | **4** | **1** | **2** | **3** | **4** | **1** | **2** | **3** | **4** | **1** | **2** | **3** | **4** | **1** | **2** | **3** | **4** | **1** | **2** | **3** | **4** | **1** | **2** | **3** | **4** |
| Metabolism and nutrition disorders | Anorexia | 1 | 0 | 0 | 0 | 1 | 1 | 0 | 0 | 2 | 1 | 0 | 0 | 3 | 0 | 0 | 0 | 2 | 0 | 0 | 0 | 0 | 0 | 1 | 0 | 9 | 2 | 1 | 0 |
| Musculoskeletal and connective tissue disorders | Joint pain | 0 | 0 | 0 | 0 | 0 | 0 | 0 | 0 | 0 | 0 | 0 | 0 | 0 | 1 | 0 | 0 | 0 | 0 | 0 | 0 | 0 | 0 | 0 | 0 | 0 | 1 | 0 | 0 |
|  | Back pain | 0 | 0 | 0 | 0 | 0 | 0 | 0 | 0 | 0 | 0 | 0 | 0 | 0 | 0 | 0 | 0 | 0 | 1 | 0 | 0 | 0 | 0 | 0 | 0 | 0 | 1 | 0 | 0 |
| Skin and subcutaneous tissue disorders | Rash | 1 | 2 | 0 | 0 | 1 | 0 | 0 | 0 | 2 | 2 | 0 | 0 | 0 | 0 | 0 | 0 | 1 | 0 | 0 | 0 | 1 | 0 | 0 | 0 | 6 | 4 | 0 | 0 |
|  | Alopecia | 0 | 0 | 0 | 0 | 0 | 0 | 0 | 0 | 1 | 0 | 0 | 0 | 1 | 1 | 0 | 0 | 0 | 0 | 0 | 0 | 0 | 1 | 0 | 0 | 2 | 2 | 0 | 0 |
| General disorders and administration site conditions | Fever | 1 | 0 | 0 | 0 | 0 | 0 | 0 | 0 | 0 | 0 | 0 | 0 | 0 | 0 | 0 | 0 | 0 | 0 | 0 | 0 | 0 | 0 | 0 | 0 | 1 | 0 | 0 | 0 |
|  | Fatigue | 1 | 0 | 0 | 0 | 2 | 0 | 0 | 0 | 1 | 2 | 0 | 0 | 1 | 1 | 0 | 0 | 3 | 2 | 0 | 0 | 2 | 1 | 0 | 0 | 10 | 6 | 0 | 0 |
|  | Pain | 0 | 0 | 0 | 0 | 0 | 0 | 0 | 0 | 0 | 0 | 0 | 0 | 0 | 0 | 0 | 0 | 1 | 0 | 0 | 0 | 0 | 0 | 0 | 0 | 1 | 0 | 0 | 0 |
|  | Edema limbs | 0 | 0 | 0 | 0 | 0 | 0 | 0 | 0 | 0 | 0 | 0 | 0 | 0 | 0 | 0 | 0 | 0 | 0 | 0 | 0 | 0 | 1 | 0 | 0 | 0 | 1 | 0 | 0 |
| Nervous system disorders | Palmar-plantar erythrodysesthesia syndrome | 0 | 0 | 0 | 0 | 0 | 0 | 0 | 0 | 0 | 0 | 0 | 0 | 0 | 0 | 0 | 0 | 0 | 1 | 0 | 0 | 0 | 1 | 1 | 0 | 0 | 2 | 1 | 0 |
|  | Peripheral sensory neuropathy | 0 | 0 | 0 | 0 | 1 | 0 | 0 | 0 | 4 | 2 | 0 | 0 | 3 | 0 | 0 | 0 | 1 | 0 | 0 | 0 | 1 | 2 | 0 | 0 | 10 | 4 | 0 | 0 |
| Gastrointestinal disorders | Constipation | 0 | 0 | 0 | 0 | 0 | 0 | 0 | 0 | 1 | 0 | 0 | 0 | 0 | 0 | 0 | 0 | 0 | 0 | 0 | 0 | 0 | 0 | 0 | 0 | 1 | 0 | 0 | 0 |
|  | Hiccups | 0 | 0 | 0 | 0 | 0 | 0 | 0 | 0 | 0 | 1 | 0 | 0 | 0 | 0 | 0 | 0 | 0 | 0 | 0 | 0 | 0 | 0 | 0 | 0 | 0 | 1 | 0 | 0 |
|  | Nausea | 4 | 5 | 0 | 0 | 5 | 4 | 2 | 0 | 9 | 11 | 1 | 0 | 7 | 4 | 2 | 0 | 9 | 6 | 1 | 0 | 2 | 3 | 1 | 0 | 36 | 33 | 7 | 0 |
|  | Abdominal pain | 0 | 0 | 0 | 0 | 0 | 0 | 0 | 0 | 0 | 0 | 0 | 0 | 0 | 0 | 0 | 0 | 0 | 1 | 0 | 0 | 0 | 0 | 0 | 0 | 0 | 1 | 0 | 0 |
|  | Diarrhea | 2 | 2 | 1 | 0 | 3 | 0 | 0 | 0 | 8 | 5 | 2 | 0 | 0 | 0 | 0 | 0 | 10 | 1 | 1 | 0 | 1 | 1 | 0 | 0 | 24 | 9 | 4 | 0 |
|  | Mucositis oral | 0 | 1 | 0 | 0 | 0 | 1 | 0 | 0 | 0 | 0 | 1 | 0 | 0 | 1 | 0 | 0 | 0 | 2 | 0 | 0 | 0 | 0 | 0 | 0 | 0 | 5 | 1 | 0 |
|  | Vomit | 0 | 4 | 0 | 0 | 3 | 2 | 0 | 0 | 3 | 7 | 3 | 0 | 1 | 4 | 1 | 0 | 4 | 1 | 0 | 0 | 4 | 0 | 1 | 0 | 15 | 18 | 5 | 0 |
|  | Gastric hemorrhage | 0 | 0 | 0 | 0 | 0 | 0 | 0 | 0 | 0 | 0 | 0 | 0 | 0 | 0 | 0 | 0 | 1 | 0 | 0 | 0 | 0 | 0 | 0 | 0 | 1 | 0 | 0 | 0 |
|  | Stomach pain | 0 | 0 | 0 | 0 | 0 | 0 | 0 | 0 | 0 | 0 | 0 | 0 | 0 | 0 | 0 | 0 | 1 | 0 | 0 | 0 | 0 | 0 | 0 | 0 | 1 | 0 | 0 | 0 |
|  | Dyspepsia | 0 | 0 | 0 | 0 | 0 | 0 | 0 | 0 | 0 | 0 | 1 | 0 | 0 | 0 | 0 | 0 | 0 | 0 | 0 | 0 | 0 | 1 | 0 | 0 | 0 | 1 | 1 | 0 |
| Vascular disorders | Hypertension | 0 | 0 | 0 | 0 | 0 | 0 | 0 | 0 | 0 | 0 | 0 | 0 | 0 | 0 | 0 | 0 | 1 | 0 | 0 | 0 | 0 | 0 | 0 | 0 | 1 | 0 | 0 | 0 |
| Blood and lymphatic system disorders | Anemia | 0 | 0 | 0 | 0 | 0 | 0 | 0 | 0 | 2 | 0 | 0 | 0 | 1 | 0 | 0 | 0 | 2 | 1 | 2 | 0 | 2 | 0 | 0 | 2 | 6 | 1 | 2 | 1 |
| Investigations | ALP increased | 0 | 0 | 0 | 0 | 0 | 0 | 0 | 0 | 0 | 0 | 0 | 0 | 0 | 0 | 0 | 0 | 1 | 0 | 1 | 0 | 0 | 0 | 0 | 0 | 1 | 0 | 1 | 0 |
|  | AST increased | 1 | 0 | 0 | 0 | 0 | 0 | 0 | 0 | 0 | 0 | 0 | 0 | 0 | 0 | 0 | 0 | 1 | 0 | 0 | 0 | 1 | 1 | 0 | 0 | 3 | 1 | 0 | 0 |
|  | CPK increased | 0 | 0 | 0 | 0 | 0 | 0 | 0 | 0 | 0 | 0 | 0 | 0 | 0 | 0 | 0 | 0 | 0 | 0 | 0 | 1 | 0 | 0 | 0 | 0 | 0 | 0 | 0 | 1 |
|  | GGT increased | 0 | 0 | 0 | 0 | 0 | 0 | 0 | 0 | 0 | 0 | 0 | 0 | 0 | 0 | 0 | 0 | 0 | 0 | 1 | 0 | 0 | 0 | 0 | 0 | 0 | 0 | 1 | 0 |
|  | White blood cell decreased | 2 | 2 | 0 | 0 | 2 | 2 | 2 | 1 | 2 | 6 | 4 | 2 | 4 | 5 | 1 | 2 | 5 | 4 | 1 | 0 | 0 | 1 | 3 | 1 | 15 | 20 | 11 | 6 |
|  | Red blood cell decreased | 0 | 1 | 1 | 0 | 0 | 0 | 0 | 0 | 1 | 0 | 0 | 0 | 0 | 0 | 1 | 0 | 0 | 0 | 0 | 0 | 0 | 0 | 0 | 0 | 1 | 1 | 2 | 0 |
|  | Hypokalemia | 0 | 0 | 0 | 0 | 0 | 0 | 0 | 0 | 0 | 0 | 0 | 0 | 0 | 0 | 0 | 0 | 1 | 0 | 0 | 0 | 0 | 0 | 0 | 0 | 1 | 0 | 0 | 0 |
|  | Platelet count decreased | 0 | 0 | 0 | 0 | 0 | 1 | 2 | 0 | 3 | 0 | 2 | 2 | 0 | 0 | 2 | 0 | 0 | 1 | 1 | 0 | 0 | 0 | 0 | 1 | 3 | 2 | 7 | 3 |
|  | Neutrophil count decreased | 0 | 6 | 3 | 2 | 1 | 0 | 4 | 2 | 2 | 6 | 4 | 0 | 2 | 2 | 5 | 2 | 2 | 7 | 2 | 1 | 2 | 2 | 2 | 2 | 9 | 23 | 20 | 9 |
|  | Blood bilirubin increased | 0 | 0 | 0 | 0 | 0 | 0 | 0 | 0 | 0 | 0 | 0 | 0 | 0 | 0 | 0 | 0 | 1 | 0 | 0 | 0 | 0 | 0 | 0 | 0 | 1 | 0 | 0 | 0 |

ALP: Alkaline phosphatase; AST: Aspartate aminotransferase; CPK: creatine phosphokinase; GGT: gamma glutamyltransferase
